# Direct Socialization of Suicide Risk in Adolescent Friendships Is Moderated by Non-Suicidal Self-Injury

**DOI:** 10.3390/bs16060843

**Published:** 2026-05-24

**Authors:** Trevor J. Long, Devan A. Walter, Abigail J. Luce, Rebecca A. Schwartz-Mette

**Affiliations:** 1 Department of Psychology, University of Maine, 300 Williams Hall, Orono, ME 04469, USA; trevor.long@maine.edu; 2 Department of Psychology, University at Buffalo, 225 Park Hall, Buffalo, NY 14260, USA; devanwal@buffalo.edu (D.A.W.); aluce@buffalo.edu (A.J.L.)

**Keywords:** adolescence, friendship, peer influence, suicide risk, NSSI

## Abstract

Given the importance of friendships and the increased risk for suicide during adolescence, potential socialization of suicidality among peers is essential to examine. Data were obtained from 93 friendship dyads (*N* = 186) in a community-based, longitudinal study of adolescents (Mage = 15.68, SD = 1.49, 69.9% female, 86.6% white). Adolescents’ and friends’ suicide risk and frequency of non-suicidal self-injury (NSSI) were assessed at baseline and at 3-month and 6-month follow-up assessments. Cross-lagged, Actor–Partner Interdependence Models (CL-APIM) examined socialization effects over time with the nested, dyadic data. Results indicated that direct socialization of suicide risk did not occur within the whole sample. However, socialization of suicide risk was observed for friends of adolescents with a past-year history of NSSI. The findings underscore the potential for NSSI to function as a susceptibility marker for socialization of suicide risk within adolescent friendships. Current study strengths, limitations, and clinical implications are further discussed.

## 1. Direct Socialization of Suicide Risk in Adolescent Friendships Is Moderated by Non-Suicidal Self-Injury

Adolescence is a developmental period in which risk for self-destructive behaviors, such as suicidal behaviors, is elevated. Myriad risk factors have been identified, including biological, cognitive, and social influences (Nock, 2009; Nock & Prinstein, 2004). One social factor receiving increased attention is peer influence. Indeed, peers appear to influence one another with regard to risk for a variety of health outcomes (Brechwald & Prinstein, 2011), including suicidal behavior (Insel & Gould, 2008). Despite documentation of suicide clusters (Gould et al., 1989), few studies have addressed whether risk for suicide may be directly socialized from peer to peer, for example, within close friendships. What is more, little is known about the conditions under which socialization of suicide risk may be facilitated. The current study examines socialization of suicide risk within adolescent friendship dyads and whether an adolescent’s history of non-suicidal self-injury (NSSI) confers additional risk for socialization to occur.

### 1.1. Peer Influence in Adolescence

Research across disciplines (including psychology, sociology, public health, political science, criminology, and marketing) acknowledge peer influence as a powerful phenomenon shaping adolescents’ lives (Prinstein & Dodge, 2008). At its core, peer influence reflects the phenomenon of homophily, i.e., the tendency of individuals to affiliate with others to whom they are similar (Lazarsfeld & Merton, 1954). Two mechanisms are thought to underlie homophily: selection and socialization (Kandel, 1978). Selection refers to the notion that individuals befriend peers who are similar to them, and socialization refers to the process(es) through which peers become more similar to one another over time.

Peer influence has been documented for many behaviors and across many contexts in adolescence including deviant or externalizing behaviors (e.g., Dishion et al., 2001), body image and disordered eating (e.g., Brechwald & Prinstein, 2011), sexual behavior (e.g., Billy & Udry, 1985), alcohol use (e.g., Hawkins et al., 1992), marijuana use (e.g., Wills & Cleary, 1999), nicotine use (e.g., Conrad et al., 1992), and internalizing symptoms like anxiety and depression (e.g., Schwartz-Mette & Rose, 2012). Past studies also suggest the influence of peers with regard to adolescent suicide (Feigelman & Gorman, 2008).

### 1.2. Socialization of Suicide Risk

Suicide is a pervasive, concerning phenomenon studied by mental health and public health experts around the world. In the US, suicide rates have increased by 30% between 2000 and 2020, and suicide is the second and third leading cause of death for youth aged 10–14 and 15–19 years, respectively (Centers for Disease Control and Prevention [CDC], 2024; Garnett et al., 2022). Risk increases across adolescence, as youth aged 15–24 die by suicide at 10x the rate of youth 10–14 (Kochanek et al., 2019). Despite efforts to improve understanding of risk for and prevention of suicidal thoughts and behaviors (STBs), a review by Franklin et al. (2017) found that studies conducted over the previous 50 years consistently identified similar risk factors with small effect sizes, underscoring the need to continue to push our understanding, particularly in understudied areas. One area of increasing focus is social influence and its role in STBs.

Interest in social influence began in the early 20th century with the documentation of suicide clusters (Bakwin, 1957; Popow, 1911), defined as an excessive number of suicides in close temporal and/or geometric proximity (Gould et al., 1989). Scholars have highlighted social transmission (indirect or direct exposure to suicide; Gould & Davidson, 1988) as a potential mechanism of influence, noting that individual and environmental factors may exacerbate or mitigate the impact of mere exposure to suicide (Gould, 1990; Haw et al., 2013). Beyond retroactive documentation of clusters, most studies of social influence in suicide focus on large-scale, indirect media influence (i.e., social media). Recent research has shown that when individuals made suicide-related posts on the social media platform X (previously Twitter), followers were then likely to make similar posts (Cero & Witte, 2020). Additionally, studies suggest that youth who view nonfictional (e.g., Romer et al., 2006) or fictional (e.g., Niederkrotenthaler et al., 2019) suicide stories in the media may be at greater risk for suicide themselves.

Fewer studies have addressed the direct (i.e., person to person) influence of peers in suicide (e.g., T. E. Joiner, 2003). Research generally demonstrates positive concurrent associations between peers’ suicidal behavior and adolescents’ own suicidal behavior (Feigelman & Gorman, 2008; Ho et al., 2000; Insel & Gould, 2008; Randall et al., 2015). Moreover, large-scale, national survey studies find an increased risk of STBs for youth exposed to peers’ suicidal behavior (Abrutyn & Mueller, 2014; Bearman & Moody, 2004; Feigelman & Gorman, 2008; Mueller & Abrutyn, 2015; Randall et al., 2015; Swanson & Colman, 2013; c.f., Harris & Bettiol, 2017). Yet these studies relied only on youths’ reports of their peers’ behavior, did not confirm the reciprocity and/or nature of the peer relationships, and could not isolate selection versus socialization effects. In fact, no studies were identified that tested direct socialization of suicide risk within close adolescent friendships. Given the importance of close friendships during the critical developmental period of adolescence (Prinstein & Giletta, 2020) and research suggesting adolescents may be more susceptible than adults to suicide contagion (Gould et al., 1990), it is essential to examine whether suicide risk might be socialized within these close relationships.

What is more, if direct socialization occurs between adolescent friends, prevention and intervention efforts would be better positioned for maximal benefit if we understood the conditions under which peer socialization is likely to occur. Past research has identified stressful life events (Harris & Bettiol, 2017), cumulative adversity (Mitchell et al., 2021), and minoritized identities (Mitchell et al., 2021) as factors influencing suicide socialization, as well as exposure to suicide-related media (Gould, 2001), family history of suicide (Ali et al., 2011), and/or another person’s death by suicide (Abrutyn & Mueller, 2014). Beyond these, little is known about factors that may facilitate the socialization of suicide risk between close friends in adolescence (Haw et al., 2013). The current study draws from relevant theories of self-injurious behavior (Nock, 2009), as well as interpersonal theories of suicidal behavior (T. Joiner, 2005; Van Orden et al., 2010), in testing one putative moderator of direct suicide socialization, non-suicidal self-injury (NSSI).

### 1.3. The Role of NSSI in Socialization of Suicide Risk

NSSI, defined as a behavior that directly damages one’s body without intent to die (Nock, 2010), is prevalent in adolescence (Swannell et al., 2014). NSSI is associated with increased STBs (e.g., Monto et al., 2018; Muehlenkamp & Gutierrez, 2007), and these associations may be understood from at least two vantage points relevant to the current inquiry. First, NSSI and STBs may have shared risk factors, such as emotional distress and diagnosed psychopathology, that contribute to their co-occurrence (Grandclerc et al., 2016; Hamza et al., 2012). Research indicates that many adolescents employ NSSI as an emotion regulation strategy that reduces emotional distress via negative reinforcement processes (Klonsky, 2007; Nock, 2009) and that psychiatric disorders are highly likely to underlie adolescents’ engagement in both NSSI and STBs (Nock et al., 2006). Second, within the framework of the interpersonal theory of suicide (T. Joiner, 2005), NSSI is thought to play a unique role in increasing risk for suicide in that it represents an acquired capability for self-destruction that, over time, results in reduced sensitivity to self-preservation instincts. This acquired capability, along with sociocognitive risk factors such as the belief that one is a burden to and/or disconnected from others, is associated with a greater likelihood of suicide attempt (Van Orden et al., 2010). Thus, an adolescent with a history of NSSI may be more vulnerable to experiencing socialization of suicide risk from friends because of their own underlying psychological distress and/or desensitization to self-destruction.

Adolescents’ engagement in NSSI also may serve an interpersonal function that impacts their friends’ sensitivity to socialization of suicide risk through modeling or social learning. NSSI may be visible and/or disclosed to close friends (Nock & Prinstein, 2004), and emerging evidence suggests that NSSI itself may be socialized between friends (e.g., Prinstein et al., 2010; Schwartz-Mette & Lawrence, 2019), perhaps in part due to this visibility or disclosure. NSSI may be more easily observed by friends than adolescents’ suicidal thinking or suicide plans, and NSSI may be more frequently observed than adolescents’ suicide attempts. Thus, observing adolescents’ NSSI may model self-destructive behavior to friends, perhaps reinforcing some level of possibility (self-efficacy) and acceptability (outcome expectancy), both of which have been linked with adolescent NSSI (Tatnell et al., 2014). The current study considers both possibilities: whether an adolescent’s past-year history of engagement in NSSI facilitates direct socialization of suicide risk from friends to adolescents and/or from adolescents to friends.

### 1.4. The Current Study and Hypotheses

The current study addressed limitations of previous research examining peer influence on suicidal risk. In a community-based, longitudinal sample of adolescent close friends, both adolescents and friends reported baseline and follow-up measures of suicide risk, allowing for analyses of dyadic data to test selection and socialization effects. Additionally, adolescents chose their close friends during the recruitment process, allowing for examination of what they considered their most influential friendship. Finally, the current study examined data gathered across three assessments over a 6-month time period, allowing for the testing of socialization effects over 3 months and 6 months.

It was hypothesized that adolescents and friends would be similar to one another with regard to suicide risk at baseline (selection effect) and that friends’ baseline suicide risk would predict increases in adolescents’ own suicide risk over time, over and above adolescents’ and friends’ baseline risk levels (direct socialization effect). Additionally, it was hypothesized that a past-year history of NSSI would moderate this direct socialization of suicide risk between adolescent close friends. Regarding NSSI moderation effects, two hypothesized possibilities were considered. We expected that, due to NSSI reflecting both emotional distress and acquired capability, adolescents’ own history of NSSI may facilitate socialization of suicide risk from friends to adolescents. Additionally, from the perspective of social modeling within friendships, we expected that adolescents’ own history of NSSI may further facilitate socialization of suicide risk from adolescents to their friends.

## 2. Methods

### 2.1. Participants

The sample consisted of adolescents and their close or best friends, aged 12 to 19 years, recruited from rural communities surrounding a mid-sized public university in New England. Target adolescents (*n* = 93) were initially recruited and then identified a same-gender friend with whom they wanted to participate, resulting in 186 total participants (Mage = 15.68, SD = 1.49; 69.9% female). All youth were enrolled in middle or high school. Participants’ self-reported racial and ethnic identities were representative of the surrounding community: 86.6% White, 4.3% Black/African American, 2.7% Asian/Pacific Islander, 1.1% American Indian/Alaskan Native, 5.4% more than one race, 3.2% Hispanic/Latino(a).

### 2.2. Procedures

All study procedures were approved by the University of Maine Institutional Review Board (IRB; #2015-10-01). The study was advertised to the community as a project aimed at understanding the impact of friendships on mental health, with planned analyses examining the reciprocal impacts of mental health symptoms and friendship functioning, as well as socialization effects of psychopathology symptoms within friendship dyads. Study hypotheses were not preregistered. Invitations were distributed via social media, public posting, and local school and community events. To be included, target participants were required to be between the ages of 13 and 17 and able to identify a same-gender friend within two years of their age. Parental informed consent for target adolescents under 18 years old was obtained, while participants 18 or older provided their own written consent. Parental informed consent was then obtained for each friend, and dyads were scheduled to attend a lab session together.

During the lab session, participants under the age of 18 provided youth assent. Adolescents and friends both completed self-report measures on computers in separate rooms. Additional measures were administered online to all participants at 3- and 6-month intervals following the lab visit. All friendships were confirmed as reciprocal at baseline (i.e., each dyad member reported that the other person was a “best” or “close” friend), and all friendships were confirmed as intact at each follow-up (i.e., no participants reported friendship dissolution). Participants each received $40 for the lab visit and $10 for each follow-up survey.

### 2.3. Measures

Suicide Risk: Participants completed the Suicide Behaviors Questionnaire–Revised (SBQ-R; Osman et al., 2001), which includes four items assessing thoughts of suicide, suicide plans, suicidal communication, and suicidal intent over the past year. Participant responses are summed to create a suicide risk score (Time 1 α = 0.84; Time 2 α = 0.75; Time 3, α = 0.80). Instructions for the Time 2 and Time 3 assessments were altered slightly to direct participants to respond with regard to their experiences since the last study assessment (e.g., Schwartz-Mette et al., 2023). As such, the Time 1 suicide risk score reflected past-year risk, and the Time 2 and Time 3 risk scores reflected risk over 3-month intervals following the Time 2 and Time 3 assessments, respectively.

Non-Suicidal Self-Injury (NSSI): At Time 1, participants rated six items assessing the frequency of engagement in non-suicidal, self-injurious behavior in adolescence (Prinstein et al., 2008). The first item examines the frequency that participants engaged in any type of NSSI in the previous year, whereas items 2–5 assess the frequency of participants engaged in specific types of NSSI in the past year (i.e., cutting, hitting, pulling hair, burning skin). Each item was rated on a 6-point Likert scale ranging from 0 (Never) to 5 (Once a day). The first item was used to identify those participants who engaged in NSSI within the past year (0 = no past-year history of NSSI, 1 = past-year history of NSSI). Because individuals with less frequent NSSI may not endorse having NSSI history on simple or single-item measures (Lund et al., 2018), participants’ responses to items 2–5 were then reviewed to ensure that any participant who reported no history of engaging in any type of NSSI (item 1) but reported a history of any specific form(s) of NSSI (items 2–5) was classified appropriately as having a past-year history of NSSI.

## 3. Results

### 3.1. Data Analysis Approach

Some participants had missing data at Time 2 and/or Time 3. Representative analyses compared the groups of participants who completed all three assessments (*n* = 103), were missing only Time 2 data (*n* = 46), were missing only Time 3 data (*n* = 76), or were missing both Time 2 and 3 data (*n* = 39) for potential mean-level differences in study variables. Groups did not significantly differ with regard to suicide risk at Time 1 [F(3, 182) = 1.52, *p* = 0.21], Time 2 [F(1, 131) = 3.31, *p* = 0.07], or Time 3 [F(1, 100) = 0.77, *p* = 0.38] or with regard to NSSI at Time 1 [F(3, 182) = 0.57, *p* = 0.64]. Little’s test further indicated that data were missing completely at random (MCAR; χ2(8) = 14.15, *p* = 0.08), and missing data were thus imputed using an expectation maximization procedure in SPSS 28.0.

Intraclass correlation coefficients estimated the degree of similarity between friends regarding suicide risk at each time point (Time 1 = 0.52, Time 2 = 0.24, Time 3 = 0.36). Primary hypotheses thus were tested using a cross-lagged, Actor–Partner Interdependence Model (CL-APIM; Cook & Kenny, 2005) to capture all three waves of data and account for the interdependence in the nested, dyadic data. The exogenous covariance between adolescents’ and friends’ Time 1 suicide risk scores was included in the model to control for initial homophily between dyad members (selection effect) and parallel endogenous covariances were added at Time 2 and Time 3. The model further included within-person actor effects represented by paths from Time 1 to Time 2 suicide risk and from Time 2 to Time 3 suicide risk for both members of the dyad. Partner effects were represented by cross paths from one friend’s Time 1 suicide risk to the other friend’s Time 2 suicide risk and from one friend’s Time 2 suicide risk to the other friend’s Time 3 suicide risk. Multiple group comparisons were then conducted to test whether the model functioned differently for adolescents with and without a past-year history of NSSI at Time 1. Although varied suggestions exist for estimating the minimum sample size required to obtain adequate power for structural equation models (Wolf et al., 2013), commonly accepted suggestions of obtaining 5–10 (Bentler & Chou, 1987) or 10–20 participants (Kline, 2010) per each of the unique parameters to be estimated in the primary hypothesized model underscored that the sample size was acceptable.

### 3.2. Descriptives and Correlations

Means, standard deviations, and bivariate correlations among all study variables are presented in Table 1. NSSI frequency was positively correlated with suicide risk scores at each time point (all *p*s ≤ 0.001). Similarly, there were significant positive correlations among suicide risk measured at each time point (all *p*s < 0.001), indicating stability in risk over time.

### 3.3. Suicide Risk Socialization

**Figure 1:**
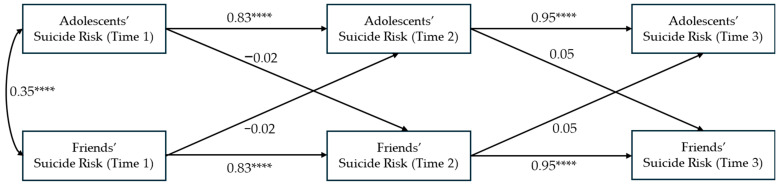
Suicide risk socialization model of whole sample over 6 months. Time 1 to Time 2 and Time 2 to Time 3 intervals were 3 months. **** *p* < 0.0001. Actor and partner effects for adolescents and friends are identical, as per the constraints of the Actor–Partner Interdependence Model for indistinguishable dyads (Olsen & Kenny, 2006).

The CL-APIM model testing direct socialization of suicide risk (see Figure 1) had excellent fit [χ2(2) = 3.44, *p* = 0.18; CFI = 1.00, TLI = 0.99, RMSEA = 0.06]. Adolescents’ and friends’ suicide risk scores were significantly and positively correlated at Time 1 (selection effect). With regard to within-person, actor effects, participants’ suicide risk scores were positively associated from Time 1 to Time 2 and from Time 2 to Time 3, suggesting stability of risk scores across waves of assessment. However, the partner (socialization) effects across time points were not significant. Specifically, adolescents’ Time 1 suicide risk did not significantly predict changes in friends’ risk at Time 2, and adolescents’ Time 2 suicide risk scores did not significantly predict changes in friends’ risk at Time 3.

### 3.4. Adolescents’ NSSI as a Moderator

**Figure 2:**
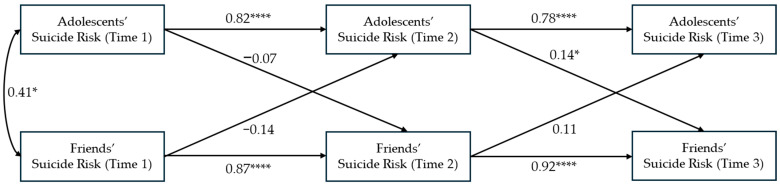
Suicide risk socialization model for adolescents with NSSI. Only individuals with a history of NSSI within the previous year are included in this model. Time 1 to Time 2 and Time 2 to Time 3 intervals were 3 months. * *p* < 0.05. **** *p* < 0.0001.

**Figure 3:**
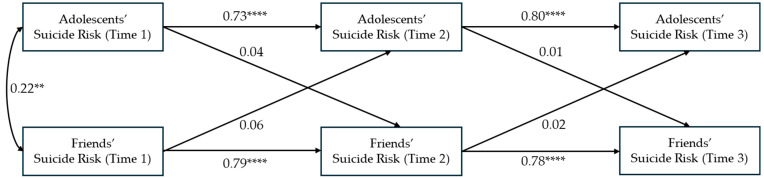
Suicide risk socialization model for adolescents without NSSI. Only individuals without NSSI within the previous year are included in this model. Time 1 to Time 2 and Time 2 to Time 3 intervals were 3 months. ** *p* < 0.01. **** *p* < 0.0001.

Next, multiple group comparisons were conducted to examine whether adolescents’ past-year engagement in NSSI moderated the socialization of suicide risk in friendship dyads over a 6-month period (see Figure 2 and Figure 3). Specifically, an unconstrained model, in which all parameters were allowed to vary by whether or not adolescents had a history of NSSI, was compared to a series of increasingly constrained models, including the structural weights (structural weights constrained to be equal across groups), structural intercepts (intercepts are constrained in addition to structural weights), structural means (means are constrained in addition to structural weights and intercepts), structural covariances (covariances are also constrained), and structural residuals (all parameters are constrained) models. The unconstrained model significantly differed from the structural weights model (Δχ2(8) = 19.58, *p* = 0.01), indicating significant differences in actor and partner effects for adolescents with and without a past-year history of NSSI.

For adolescents who had engaged in NSSI in the past year (*n* = 42), stability of their suicide risk scores was observed from Time 1 to Time 2, and from Time 2 to Time 3. Significant actor/stability effects also were observed for friends of adolescents who had a history of NSSI. In contrast to hypotheses, socialization effects from friends to adolescents were not observed for adolescents with a history of NSSI. Interestingly, socialization effects were observed for the friends themselves. Specifically, for adolescents with a history of NSSI, their suicide risk scores at Time 2 significantly predicted increases in their friends’ suicide risk at Time 3.

For adolescents who did not engage in NSSI in the past year (*n* = 144), significant stability effects were observed for adolescents and their friends. Notably, for adolescents who did not engage in NSSI in the past year, no socialization effects were observed from adolescents to friends or from friends to adolescents. Taken together, results suggest that socialization effects for suicide risk were significant only for friends of adolescents with a history of NSSI in the previous year.

## 4. Discussion

Adolescence involves significant developmental transitions and increased risk for maladjustment, and peers often serve as models and function as the most influential members of one’s social environment during this time (Steinberg, 1990). Using a community sample and longitudinal design, the current exploratory study estimated adolescents’ and friends’ initial similarity in suicide risk (i.e., selection effect) and tested whether friends’ initial suicide risk was predictive of adolescents’ later suicide risk (i.e., direct socialization effect) over 3- and 6-month intervals within reciprocal, close friend dyads. Additionally, the current study explored whether adolescents’ past-year history of NSSI moderated the direct socialization of suicide risk and in what direction this socialization may occur–from adolescents to friends and/or from friends to adolescents.

Results suggested that, contrary to hypotheses, suicide risk was not directly socialized in the whole sample of adolescent close friends during the time periods studied. These results were consistent with previous work suggesting that mere exposure to another’s suicidality is unlikely to create suicide risk, especially where none existed previously (Gould & Davidson, 1988). Indeed, examination of adolescents’ and friends’ similarity in suicide risk scores at baseline underscore that youth tend to form friendships with those to whom they are similar (i.e., selection effects). It is possible that, given the relatively low mean levels of suicide risk across the sample, future studies of clinical populations may be better powered to detect direct socialization effects. Moreover, assessment of imminent suicide risk and family support (e.g., referral information) was provided to adolescents who reported a recent suicide plan or attempt at each time point. Although important for safety, it is possible that this resulted in adolescents altering their response style (e.g., underreporting), which is a limitation of the current study. Another important limitation to consider is that suicide risk data were constrained to STBs that had occurred in the past year. This means that direct socialization of lifetime suicide risk could not be examined in the current study.

A lack of observed direct socialization does, however, underscore the potential importance of examining socialization under conditions of enhanced vulnerability. Consistent with research suggesting that exposure to peers’ suicide risk may increase risk for vulnerable populations (Lake & Gould, 2014), results from the current study suggest that friends of adolescents with a past-year history of NSSI may be vulnerable to socialization of suicide risk. Specifically, direct socialization of suicide risk was observed for friends of adolescents who had recently engaged in NSSI. The socialization effect was significant, even after controlling for selection (i.e., initial similarity between dyad members) and friends’ own baseline suicide risk. Given the relatively small subgroup of participants who reported NSSI history, it will be important for future research to replicate this effect to enhance confidence in these results.

Why might adolescents’ NSSI be a condition under which friends experience increased suicide risk? From a social cognitive or social learning perspective (Bandura, 1986), one avenue by which youth adopt new behaviors is modeling. Research suggests that modeling may be especially powerful when youth respect, like, and identify strongly with the model (e.g., Heilbron & Prinstein, 2010). The very nature of friendships as egalitarian, voluntary, and affiliative underscores why friends are such powerful socialization agents at this stage (Prinstein & Dodge, 2008), and emerging theory focused exclusively on peer influence sheds additional light on why adolescents may be especially motivated to adopt peers’ maladaptive behaviors. In particular, the Influence-Compatibility Model (Laursen & Veenstra, 2021) posits that the primary function of peer influence is to increase similarity among affiliated partners in order to promote compatibility, reduce conflict, and avoid social rejection.

Being friends with an adolescent who self-injures may expose the friend to visual and/or verbal information about self-destructive behaviors. For example, friends may view evidence of adolescents’ NSSI (e.g., current wounds, scars) or discuss the adolescents’ NSSI with them during conversations. These friends may come to see self-destructive behaviors as increasingly possible and acceptable. Alternatively, friends who see adolescents’ NSSI may misinterpret these visuals as evidence of recent suicide attempts, enhancing modeling effects. Future research should test whether exposure to adolescents’ NSSI predicts changes in friends’ expectancies and self-efficacy regarding STBs, as well as their engagement in STBs, over time.

Because data from the current study cannot speak directly to the precise processes by which socialization of suicide risk may occur, it will be important for future research to elucidate the mechanisms by which suicide risk is socialized within adolescent friendships. Haw et al. (2013) conducted a review of studies examining mechanisms and risk factors underlying suicide clusters. Although they identified contagion, imitation, suggestion, learning, and assortative relating as potential underlying mechanisms, supporting empirical evidence is generally lacking and studies examining these mechanisms lacked methodological rigor (Haw et al., 2013). Clarifying mechanisms will enable the confirmation and/or extension of existing recommendations for impeding socialization in those in close contact with self-injuring adolescents (e.g., Richardson et al., 2012).

Previous studies suggest that individuals may talk about or even engage in NSSI together with friends (Glenn & Klonsky, 2009; Heath et al., 2009; Zelkowitz et al., 2017), and that having a friend who discloses about a suicide attempt predicts increased suicide risk for adolescents themselves. However, research is limited with regard to understanding the nuances of how adolescent friends talk together about their STBs. Theories of self-disclosure in adolescent friendships suggest that youth not only gain support and validation from such conversations with friends, but that self-disclosure serves self-clarification and social control functions as well (Buhrmester & Prager, 1995). Future observational studies may benefit form examining adolescents’ conversations in which suicidality (and NSSI) are discussed in more depth to further grasp potential underlying mechanisms of socialization. For example, excessively discussing problems with friends (co-rumination; Rose, 2002) has been shown to mediate socialization of depressive symptoms within adolescent friendships (Schwartz-Mette & Rose, 2012; Schwartz-Mette & Smith, 2018). It may be that the ways in which adolescents discuss their self-destructive urges or behaviors with friends serves a similar socialization function for suicide risk. Perhaps friends who engage in NSSI are more likely to disclose their co-occurring STBs, thus impacting adolescents’ own STBs. A recent study of adolescents in Saudi Arabia did find that friends’ disclosure of self-harm was positively associated with adolescents’ own suicidality (Copeland et al., 2021).

Future research could address other limitations of the study, which was conducted in a relatively small, community sample in which not all adolescents experienced suicide risk or NSSI. Replicating analyses in larger, more diverse samples and with adjustments to methodology would enhance confidence in and clarify results in multiple ways. First, post hoc power analyses indicated that the sample was adequately powered for detecting medium and large effects, but not small effects. White and female adolescents also were overrepresented in the sample, which limits external validity and generalizability of results. Second, because the timeframes for assessment differed at baseline (past year history of suicide risk and NSSI frequency) versus follow-ups (past 3 months), future studies should assess symptoms over equivalent timeframes. Additionally, given limited variability in the NSSI frequency variable, scores were dichotomized to reflect presence versus absence of NSSI only; future work may benefit from recruiting clinical or at-risk samples, to guarantee sufficient variability in frequency scores to retain this important information.

Third, future research with larger samples should simultaneously test direct socialization of both NSSI and STBs to isolate unique socialization effects, if present. Adding NSSI socialization paths to the current models resulted in convergence problems, likely due to sample size. It is also possible that the strong association between suicide risk scores and NSSI history may reflect baseline severity of distress or a broader psychological vulnerability. What is more, it could be that adolescent friends are influenced by a shared risk context or another, unmeasured, third variable, which could be influencing results. Larger samples would also be better powered to control for and address the role of other relevant variables (e.g., risk and resilience factors) and to explore multiple sources of influence (e.g., other friends, peer group network effects) beyond the focus on just one friend.

The current study also only examined same-gender friendships. Although a majority of adolescents’ close friendships are same-gender (Maccoby, 1990), and most of the dyadic peer influence literature focuses on same-gender pairs, adolescents do have some cross-gender friendships, and peer influence is understudied in such relationships, pointing to another important area of inquiry for future socialization work. Additionally, the current study collected participant data over the course of three time points, spanning 6 months from baseline data collection; yet significant socialization effects were found only from Time 2 (i.e., 3 months post-baseline) to Time 3 (i.e., 6 months post-baseline) in the subsample of adolescents with a history of NSSI. This initial finding raises more questions about the timing of socialization than the current data can answer. The phenomenon of homophily (Lazarsfeld & Merton, 1954), and more specifically, of socialization (Kandel, 1978), posits that friends become more similar to each other over time. On the one hand, all participating dyads were reflective of existing close or best friendships; thus, some socialization could already have occurred prior to the current study. On the other hand, it could be that socialization effects require longer than 6 months to become more prominent. Future research would benefit not only from assessing outcome measures over a longer follow-up period and more frequently (i.e., every month instead of every three months), but also from studying selection and socialization effects nearer to the beginning of friendship formation, such as at the transition to high school, to provide more precise information about when and how socialization may occur.

Despite its limitations, this work may have practical implications. Given that adolescent friendships are often formed and maintained through schools and community-based activities, adults who work with adolescents in these spaces will benefit from understanding the potential role of peer influence as it pertains to mental health. The results also underscore the potential utility of interpersonal clinical assessments. For example, mental health screening could involve questions not only about adolescents’ own mental health but also the mental health of their friends. Relatedly, clinical interventions for distressed youth may have benefits not just for those who are struggling overtly but for their friends as well. Interventions should aim to maximize protective friendship processes such as companionship and social support (Parker & Asher, 1993) while mitigating suicide risk socialization processes. Although an interpersonal behavioral mechanism for the socialization of self-destructive behavior has yet to be identified, future research could explore the way(s) that friends communicate with one another about their distress as one possibility. For example, co-rumination, a dyadic process involving excessive discussion of problems and focus on negative affect, has been found to facilitate socialization of depressive and anxiety symptoms in adolescent friendship dyads (Schwartz-Mette & Rose, 2012).

In conclusion, although the current study did not find evidence for the direct socialization of suicide risk within all close friend pairs, the findings highlight a critical nuance: the presence of recent NSSI may be one marker of enhanced susceptibility for the social transmission of suicide risks. By identifying this specific vulnerability, this research encourages ongoing work to further clarify how self-destructive behaviors may be normalized, modeled, and/or reinforced between friends. Recognizing that an adolescent’s risk may be linked to the mental health of their closest peers offers a vital pathway for more effective, system-oriented prevention and intervention strategies in school and clinical settings.

## Figures and Tables

**Table 1:** Descriptive Statistics and Correlations.

	M (SD)	1.	2.	3.	4.
Variable					
1. Time 1 NSSI	0.39 (0.88)	–			
2. Time 1 Suicide Risk	4.94 (2.78)	0.65 ***	–		
3. Time 2 Suicide Risk	4.30 (1.89)	0.58 ***	0.82 ***	–	
4. Time 3 Suicide Risk	4.25 (1.92)	0.73 ***	0.79 ***	0.84 ***	–

*** *p* < 0.001.

## Data Availability

The original contributions presented in this study are included in the article. Further inquiries can be directed to the corresponding author.
